# Selective Functional Interaction Between the Lateral Habenula and Hippocampus During Different Tests of Response Flexibility

**DOI:** 10.3389/fnmol.2019.00245

**Published:** 2019-10-15

**Authors:** Phillip M. Baker, Yingxue Rao, Zeena M. G. Rivera, Esteli M. Garcia, Sheri J. Y. Mizumori

**Affiliations:** ^1^Department of Psychology, Seattle Pacific University, Seattle, WA, United States; ^2^Department of Psychology, University of Washington, Seattle, WA, United States; ^3^Program in Neuroscience, University of Washington, Seattle, WA, United States

**Keywords:** spatial delayed alternation, delay discounting, baclofen, muscimol, rats

## Abstract

The lateral habenula (LHb) has been shown to play critical roles in a variety of appetitive tasks (e.g., spatial memory and object recognition) that require animals to flexibly respond to changing task conditions. These types of tasks are known to be dependent on hippocampus (HPC) and/or medial prefrontal cortex (mPFC), suggesting that the LHb contributes to the limbic memory circuit. Here we provide new evidence that the LHb and HPC play distinct but complimentary roles in tasks that require flexible responding to changing task conditions. Experiment 1 tested whether the LHb is needed for the performance of a HPC-dependent maze-based spatial delayed alternation task. The importance of interactions between the LHb and HPC to accomplish the same spatial delayed alternation task was tested in Experiment 2 where the LHb and HPC were disconnected both ipsilaterally and contralaterally. Experiment 3 tested LHb’s involvement in a standard behavioral economic task that requires flexible responding (maze-based delayed discounting), a task previously shown to rely on HPC. Results of Experiment 1, revealed that LHb inactivation impairs spatial delayed alternation during asymptotic performance but not during initial learning. Importantly, working memory did not appear to be affected as performance remained above chance levels both during initial learning and asymptotic testing. Experiment 2 showed that ipsilateral and contralateral disconnection of the LHb and HPC led to impaired performance on the spatial delayed alternation task. Impairments were not observed after unilateral inactivation of only one structure. Results of Experiment 3 were similar to our previous report of the effects of HPC inactivation: LHb inactivation impaired delayed discounting. All effects could not be accounted for by changes in reward magnitude discrimination, reward location *per se*, or sex of the animal. These findings, combined with other recent publications confirms and extends our working hypothesis that the LHb enables adaptive and flexible responding, particularly when established rules must be flexibly applied on a trial by trial basis. Since there are no known direct anatomical connections between LHb and HPC, future research is needed to understand how these structures communicate to enable flexible and rapid responding.

## Introduction

All animals share a common and essential need to engage behavioral adaptation strategies, i.e., the process of rapidly switching amongst learned strategies when a subjective aim or objective framework is altered (Mizumori et al., 2004; White et al., 2013; Hasson et al., 2015). Decades of prior research has implicated key roles for several brain areas in flexible response selection. Among these are the hippocampus (HPC), lateral habenula (LHb), and the medial prefrontal cortex (mPFC; Sutherland, 1982; Dalley et al., 2004; Barker and Warburton, 2013). However, a complete story of how these varied and distal brain regions interact to enable one to rapidly and dynamically switch behavioral responses is yet to develop. Recent research has implicated functional interactions between the mPFC and HPC, the mPFC and LHb, and the LHb and HPC across different types of task demands. For example, performance of an object-in-place recognition task was dependent on excitatory transmission between the HPC and mPFC (Barker and Warburton, 2013). It was also found that HPC and LHb local field potentials were coupled in the theta range and that the degree of coupling was positively correlated with performance accuracy on a spatial object recognition task (Goutagny et al., 2013).

**Figure 1:**
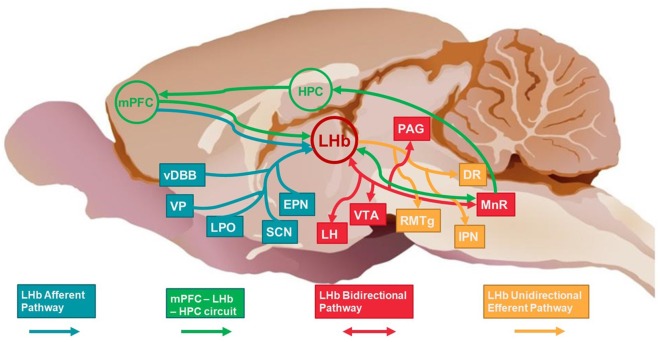
Schematic of selected afferent, bidirectional and efferent pathways of LHb (red circle) and the broader HPC-mPFC-LHb circuit. Afferent structures/pathways are shown in blue, bidirectional structures/pathways are shown in red, efferent structures/pathways are shown in yellow. The mPFC-LHb-HPC circuit structures/pathways are shown in green. LHb, lateral habenula; HPC, hippocampus; mPFC, medial prefrontal cortex; vDBB, vertical diagonal band of Broca; VP, ventral pallidum; LPO, lateral preoptic area; SCN, suprachiasmatic nucleus; EPN, entopeduncular nucleus; LH, lateral hypothalamus; VTA, ventral tegmental area; RMTg, rostromedial tegmental nucleus; IPN, interpeduncular nucleus; MnR, median raphe; DR, dorsal raphe; PAG, periaqueductal gray. Rat brain background credited to Brown (2018).

We previously proposed a model (Mizumori and Baker, 2017) that describes interactions between the HPC, LHb, and mPFC during memory dependent decision making. According to our model, the mPFC provides integrated context-specific information (determined in part through input from the HPC) to inform the LHb of the current decision-making strategy. The LHb is hypothesized to integrate mPFC afferent information with input from other brain areas (including motivational and reward structures) to determine whether the strategy is appropriate or needs to be adjusted (Mizumori and Baker, 2017). If the response is still appropriate, LHb efferent signals to the ventral tegmental area (VTA) and median raphe (MnR) promote continued responding. If there is a mismatch between mPFC signals of memory-guided response decisions and the internal state of the animal, LHb efferent signals to VTA and MnR should result in changed responses. In the latter case, LHb may indirectly inform HPC (*via* direct MnR input to HPC) when temporal and spatial sequences of context information should be updated (Figure 1).

This model suggests a complementary but unique role for the LHb during mPFC or HPC dependent tasks (Hikosaka et al., 2008; Kim and Lee, 2012; Shabel et al., 2012). Support for a complementary but distinct role is supported by prior research. For example, mPFC inactivation led to enhanced probabilistic reversal learning performance due to enhanced sensitivity to positive rewards and decreased sensitivity to negative outcomes (Dalton et al., 2016). In contrast, LHb inactivation during probabilistic reversal learning impaired performance while reducing sensitivity to both positive and negative outcomes (Baker et al., 2015). Other tasks have shown a more similar role for the mPFC and LHb in task performance. For example, in a delayed non-match to sample task pharmacological disconnection of these areas resulted in impaired performance (Mathis et al., 2016). Interestingly, similar results were seen in this task with either ipsilateral (same hemisphere) or contralateral (opposite hemisphere) disconnection.

This last result raises an important point regarding LHb influences on behaviors. Specifically, each hemispheric LHb projects bilaterally to downstream areas such as the raphe nuclei and rostromedial tegmental area (Araki et al., 1988; Sego et al., 2014; Quina et al., 2015). This bilateral projection has been proposed to be a result of selective evolutionary pressure to optimize the integration of diverse telencephalic information for binary choices, as elegantly described in (Ichijo et al., 2017). Thus, the LHb likely plays a fundamental role in behavioral selection based on current strategy information (e.g., from the mPFC) and other internal factors. In fact, there is growing support for this idea across several species (Agetsuma et al., 2010; Okamoto et al., 2012; Stephenson-Jones et al., 2016). Together, these data further support the hypothesis that the LHb and mPFC play complementary but distinct roles in behavioral selection.

Less clear is the nature of the functional interactions between the HPC and LHb during adaptive behavioral selection. Several examinations of the HPC and LHb have proposed similar roles in memory-related behaviors. As previously mentioned, the strength of theta synchrony between the HPC and LHb is related to object recognition (Goutagny et al., 2013), and Morris water maze tests reveal similar effects of LHb lesions to those observed with HPC manipulation, namely, impairment reaching the escape platform (Thornton and Davies, 1991; Riedel et al., 1999; Lecourtier et al., 2004). It is worth pointing out, however, that several recent findings indicate differing contributions of the HPC and LHb to appetitive task performance. For example, unlike with HPC manipulations, LHb inactivation immediately following learning (e.g., during consolidation) does not alter water maze performance (Micheau et al., 2004; Mathis et al., 2015). Additionally, while fimbria/fornix lesions (designed to disrupt HPC communication) do not alter reversal learning performance unless combined with mPFC lesions (Mala et al., 2015), bilateral LHb inactivation is sufficient to disrupt probabilistic reversal learning that is distinct from effects of mPFC manipulation (Baker et al., 2015). This pattern of effects suggests that while the LHb contributes to memory-related tasks, it does not exactly match the effects of HPC inactivation.

To directly test contributions of the LHb and HPC during memory-related decision-making, Experiment 1 of the present study used a spatial location-based delayed alternation task that was designed to examine the effects of LHb inactivation during learning and asymptotic performance. Specifically, *if* the LHb is required to use working memory, *then* animals should perform at chance levels due to an inability to recall the preceding arm visit during both early learning and asymptotic performance. However, *if* the LHb is required for the application of a learned rule (reference memory), *then* LHb inactivation should result in above chance performance similar to controls during early learning. However, during asymptotic performance, LHb inactivation should impair, but not reduce to chance levels, choice accuracy due to an innate preference for rats to alternate choice arms. A second experiment was then performed to determine whether disconnection of the HPC and LHb (both ipsilaterally and bilaterally) is sufficient to cause behavioral deficits as would be suggested if these areas functionally interact during task performance.

In a third experiment, we sought to clarify another issue relevant to our understanding of contributions of the HPC and LHb to memory-driven flexible responding. Previous research has shown that HPC inactivation increases the variability of choices when delays are increased on a maze based delay discounting task (Bett et al., 2015). In contrast, LHb inactivation led animals to show no preference for either the immediate or delayed reward when tested on an operant chamber version of delay discounting (Stopper and Floresco, 2014). To clarify this apparent difference between the roles of the HPC and LHb during delay discounting, we examined the effects of bilateral LHb inactivation as rats ran a maze-based version of the delay discounting task. In this way, we could determine if differences in the previous studies arose because of the use of spatial or nonspatial tests, or if they were due to fundamental differences in information processed by the LHb and HPC.

When combined, the results from these experiments further clarify the nature of the relationship between LHb and HPC processing when subjects are required to make decisions across diverse forms of behavioral flexibility.

## Materials and Methods

### General Methods

#### Animals

Experimentally naïve male Long–Evans rats (Experiment 1: *n* = 27; Experiment 2: *n* = 6; Experiment 3: *n* = 4; 320–400 g; 60–70 day old; Charles River) and female Long–Evans rats (Experiment 2: *n* = 7; Experiment 3: *n* = 4; 220–270 g; 60–70 days old, Charles River) were individually housed in a temperature-controlled laboratory (accredited by the Association for Assessment and Accreditation of Laboratory Animal Care). The room in which they were housed was maintained on a 12 h light/dark cycle (lights on at 7:00 am). Rats were allowed to free feed for a week, and then they were placed on a standard food restriction schedule so that they could gradually reach and maintain 85% of their original free feed weight. Rats were trained and tested during their light cycle (9:00 am to 5:00 pm) in accordance with the University of Washington’s Institutional Animal Care and Use Committee guidelines (Protocol 3279-01).

#### Behavioral Apparatus

##### Experiments 1 and 2

A black Plexiglas elevated cross-maze (79 cm from the floor, 58 × 5.5 cm maze arms) contained a 3D printed food well connected *via* tubing to a computer-controlled pellet delivery apparatus (Lafayette Instruments, Lafayette, IN, USA) which provided sucrose rewards (one 45 mg sucrose pellet; TestDiet, Richmond, IN, USA) at the ends of the two goal arms. The maze was controlled remotely with LabVIEW 2016 software (National Instruments, Austin, TX, USA). Each maze arm was hinged such that its proximal end (i.e., the segment closest to the maze center) could be raised and lowered by computer control. During the choice epoch, both goal arms were available; during the return epoch, only one start arm was available to guide the rat back to the start arm. A black curtain with visual cues attached surrounded the maze.

##### Experiment 3

A black Plexigas elevated T-maze (79 cm from the floor) was configured with one start arm and two goal arms. A metal food cup was located at the end of each goal arm. Wooden barriers were placed in front of each food cup to control access to the reward. Upon entering a reward arm, a second block was placed behind the rat so that it could not exit the arm during the delay. Once the specified delay time passed, the block in front of the food was lifted. When the rat finished eating, the second barrier behind the animal was lifted so that it could return to the start arm. As in Experiments 1 and 2, each maze arm was hinged such that its proximal end closest to the maze center could be raised and lowered by remote control as a way to control access to food by the rat. During forced trials, only one of the two goal arms was available to the animal. During free-choice trials, both goal arms were available. The maze was encircled by black curtains that were decorated with spatial cues.

#### Surgical Procedures

##### Experiments 1 and 3

Under anesthesia with isoflurane (4% mix with oxygen at a flow rate of 1L/min), rats were mounted in a stereotaxic instrument (David Kopf Instruments, Tujunga, CA, USA). Subsequently, the isoflurane concentration was reduced to 1%–3% to maintain the desired anesthetic depth. The skull was exposed and adjusted to place bregma and lambda on the same horizontal plane. All animals were implanted with two 25-gauge cannulae bilaterally in the LHb (anteroposterior = −3.5 mm; mediolateral = ±0.8 mm; dorsoventral = −4.5 mm, top of skull). The double cannulae were secured in place with anchoring screws and dental cement. Following implantation, 33-gauge double dummy cannulae were inserted to prevent clogging, and fitting caps were added to keep dummy cannulae in place. After surgery, all rats were given 5–7 days of surgical recovery and daily handling before postoperative training began. Rats in Experiment 1 underwent two-arm training until consistent food retrieval behavior was observed. Rats in Experiment 3 were then retrained on the delay discounting task until they reached stable, consistent discounting behavior.

##### Experiment 2

Surgical procedures occurred as described above for Experiments 1 and 3, except that rats were implanted with two 25-gauge cannulae bilaterally in the LHb (anteroposterior = −3.5 mm; mediolateral = ±0.8 mm; dorsoventral = −4.5 mm, top of skull), and two single 25-gauge cannula bilaterally in the HPC (anteroposterior = −3.9 mm; mediolateral = ±3.5 mm; dorsoventral = −2.1 mm, top of skull; with a 25-degree angle).

#### Microinjection Procedures

A day before microinjection, the injection cannula (Plastics One, Roanoke, VA, USA; which extended 1 mm beyond the guide) was inserted into the guide cannula and left in place for 1 min. This was done to control for any initial mechanical damage done by the injector, and to habituate the rat to the injection procedure. On a test day, rats were injected with drug (a combination of baclofen and muscimol in Experiments 1 and 2; only muscimol in Experiment 3; Sigma) dissolved in 0.9% saline, or vehicle. All LHb injections used a volume of 0.2 μl (50 ng/0.2 μl drug) and a 0.15 μl/min infusion rate. In Experiment 2, the HPC dose was increased to 200 ng/0.2 μl while the volume and timing remained the same. Bilateral infusion was administered to the animal 10–15 min prior to the start of the behavioral session, and all sessions were finished within 60 min after infusion. This volume and infusion rate were similar to those used in other LHb and HPC inactivation studies that infused baclofen and muscimol (Yoon et al., 2008; Stopper and Floresco, 2014; Baker et al., 2015). The injection cannula was connected to a 10 μl syringe (Hamilton) *via* polyethylene tubing (PE 20). Constant infusion rates were accomplished with the aid of an infusion pump (KD Scientific).

#### General Statistical Analyses

Using the G*Power 3 analysis program, it was determined that about seven rats per group will give statistical power at the 0.01 level. Key assumptions were that rats that achieve 80% choice accuracy have learned the task and that rats that perform 20% below controls (about 64%) are learning impaired. Data were analyzed with two-way ANOVA, one-way ANOVA, and paired sample *t*-test. Two-tailed *p*-values < 0.05 were considered statistically significant. All data are expressed as mean ± SEM.

### Behavioral Training

#### Experiment 1: Effects of LHb Inactivation During Initial Learning and Asymptotic Performance of a Spatial Delayed Alternation Task

##### Habituation and Pre-surgical Training

Over the course of 3–5 days, all rats underwent habituation to the maze wherein they were allowed to freely forage for sucrose pellets that were randomly scattered on four maze arms (two start and two goal arms). Rats were then shaped to collect a one-pellet reward from one of the two goal arms of a plus maze (with both arm available for them to enter) and return to one of the pseudo-randomly chosen start arms (two-arm training task). There was an inter-trial interval of 5 s between trials, which were defined as the successful retrieval of a reward from a goal arm and return to a start arm. Once rats finished 45 trials in under 30 min for two consecutive days, they underwent the surgical implantation of bilateral cannulae. This experiment was performed prior to the laboratory adopting the practice of including both male and female subjects and thus only includes male subjects. Due to the lack of effect of sex on task performance in the other two experiments, it was determined that additional female subjects for this experiment would not be a prudent use of animal subjects.

##### Spatial Delayed Alternation Task (Figure 2)

After a week of recovery from surgery, all rats were put back on a food-restricted diet. The 27 animals were trained on the two-arm training task to return to presurgical performance levels. Then rats were trained on the delayed alternation task. A trial consisted of the following sequence: In the start or delay epoch (10-s waiting period before the start of next trial), animals waited in one of the start arms. In the choice epoch, the extension of the start arm allowed the rat to enter the choice area to choose between the two available reward arms. Once the animal entered a goal arm, the other goal arm was lowered to signal the start of a reward epoch where the rat would retrieve one sucrose pellet if it chose the opposite reward arm from the previous trisl (repeated entrance to the same arm would result in no reward). After the animal finished eating the pellet, it could return to the next start arm (pseudo-randomly chosen by a prewritten computer algorithm designed so that there were no more than two consecutive starts from the same arm) where the inter-trial interval delay began. After the delay, the start arm was elevated so that the rat could start the next trial. Rats were tested for a total of 60 trials in a session. Prior to testing rats were randomly assigned to one of three groups that were identified by the type of first and second infusion given during respective task phases. Nine rats were assigned to each of the Sal-Sal, Drug-Sal, or Sal-Drug groups. All three groups were tested on a delayed spatial alternation task during learning and asymptotic performance phases. The first injection (e.g., Sal in the Sal-Drug group) was given prior to each of the first 3 days of testing on the delayed alternation task (*learning performance*). Subsequently, rats continued to be trained on the delayed alternation task without receiving injections until *asymptotic performance* was achieved (defined as three consecutive days at >80% accuracy). Once achieved, rats were given a further 3 days of injection (e.g., the Drug in the Sal-Drug group). Finally, once asymptotic injections were completed, rats were given a final day of testing drug free to check for any lasting effects of the treatment on performance.

#### Experiment 2: Effects of LHb and HPC Disconnection During Spatial Delayed Alternation Tests

##### Habituation and Pre-surgical Training

Because no effect of drug treatment was observed during the learning phase of Experiment 1, Experiment 2 focused on effects during asymptotic performance. After the habituation and two-arm training task described in Experiment 1, rats (*n* = 13, seven females and six males) in this experiment started the delayed alternation task training. After achieving 80% accuracy across three continuous days, cannulae were surgically implanted.

##### Spatial Delayed Alternation Task

After a week of recovery from surgery, all animals were put back on a food-restricted diet. Once presurgical delayed alternation asymptotic performance was achieved (>80% accuracy for three consecutive days), rats were assigned to receive in pseudo-random order the following set of injections: (1) ipsilateral HPC-LHb saline injection; (2) ipsilateral HPC-LHb drug injection; (3) contralateral HPC-LHb saline injection; (4) contralateral HPC-LHb drug injection; (5) unilateral LHb saline injection; (6) unilateral LHb drug injection; (7) unilateral HPC saline injection; and (8) unilateral HPC drug injection (Figure 4A). Each rat underwent all eight injections with injection sides that were balanced across left and right hemispheres. In between each injection, rats were given a recovery day without injection to ensure performance recovered from any deficits induced by treatment.

**Figure 2:**
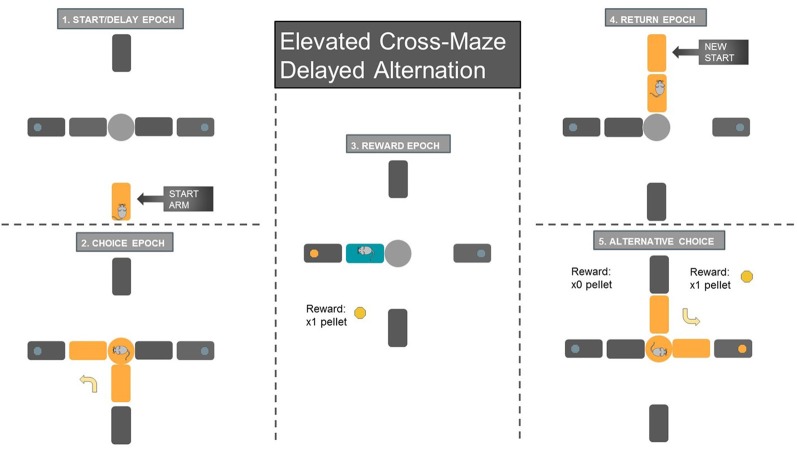
Schematic of Delayed-Alternation Task. (1) Animals waited on the start arm for 10 s during the start/delay epoch. The arm segment closest to the center platform was lowered to confine the animal to the start arm region for the delay period. (2) The choice epoch started when the animal entered the center platform of the maze and made a choice to the left/right reward arm. The blue circle at the end of each reward arm signifies the metal cup where sugar pellets were delivered through food dispensers upon the animal’s arrival. (3) The reward epoch, which was analyzed only for the rewarded choice, while the other reward or start arm was lowered. Animals consumed the sugar pellet once they obtained the reward at the metal cup. (4) The return epoch began when the animal finished reward consumption and returned to the recently raised start arm (start arm location was pseudorandomized over the 60 choice trials). (5) Once the animal finishes the delay period, the next trial began. Only alternating choices were rewarded, while repeated entrance to the same reward arm resulted in no reward. After the animal returned to the start arm and waited for the 10 s delay period, a new trial began.

**Figure 3:**
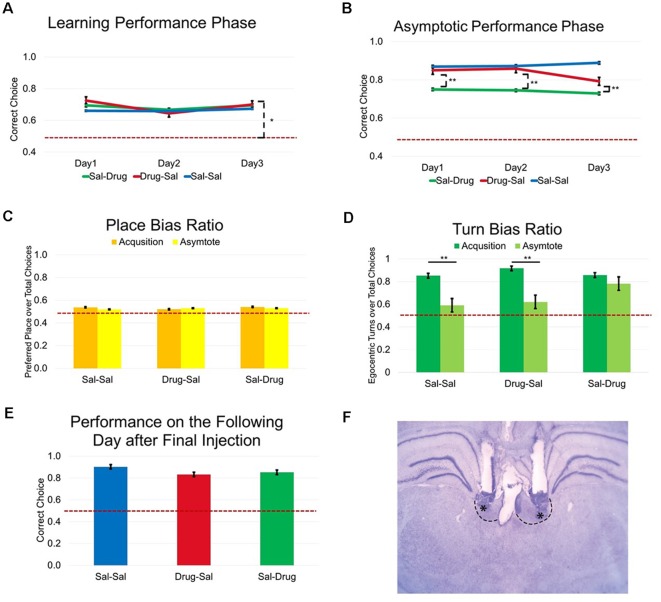
Experiment 1: LHb inactivation impairs learned spatial delayed-alternation but does not alter initial learning. **(A)** Drug treated animals are more accurate than would be expected by chance (*t*_(2)_ = 7.94, *p* < 0.05). **(B)** LHb inactivation altered flexible performance of a learned behavior performed at asymptotic levels. **(C)** No effects of treatment group (*F*_(2,16)_ = 0.80, ns.), learning phase (*F*_(1,16)_ = 1.01, ns.), or interaction (*F*_(2,16)_ = 1.55, ns.) were observed according to a two-way ANOVA on the ratio of preferred place location over the total number of choices during a session. **(D)** Examination of egocentric turn bias using a two-way ANOVA revealed a significant effect of treatment group (*F*_(2,16)_ = 5.11, *p* < 0.05), learning phase (*F*_(1,16)_ = 38.26, *p* < 0.01), and a significant interaction (*F*_(2,16)_ = 4.23, *p* < 0.05) on the ratio of preferred egocentric turn direction over the total number of choices during a session. Bonferroni *post hoc* tests showed that the turn bias ratio was significantly lower during asymptotic performance for the Sal-Sal and Drug-Sal groups but not for the Sal-Drug group (*p* < 0.01). **(E)** A one-way ANOVA revealed a similar level of performance accuracy following the final day of asymptotic testing, *F*_(2,16)_ = 3.03, ns. ranging from (80.79 ± 3.32) in the Drug-Sal group to (90.28 ± 1.69) in the Sal-Sal group. **(F)** Cannula placements in the LHb. *p < 0.05. **p < 0.01. ns = not significant.

**Figure 4:**
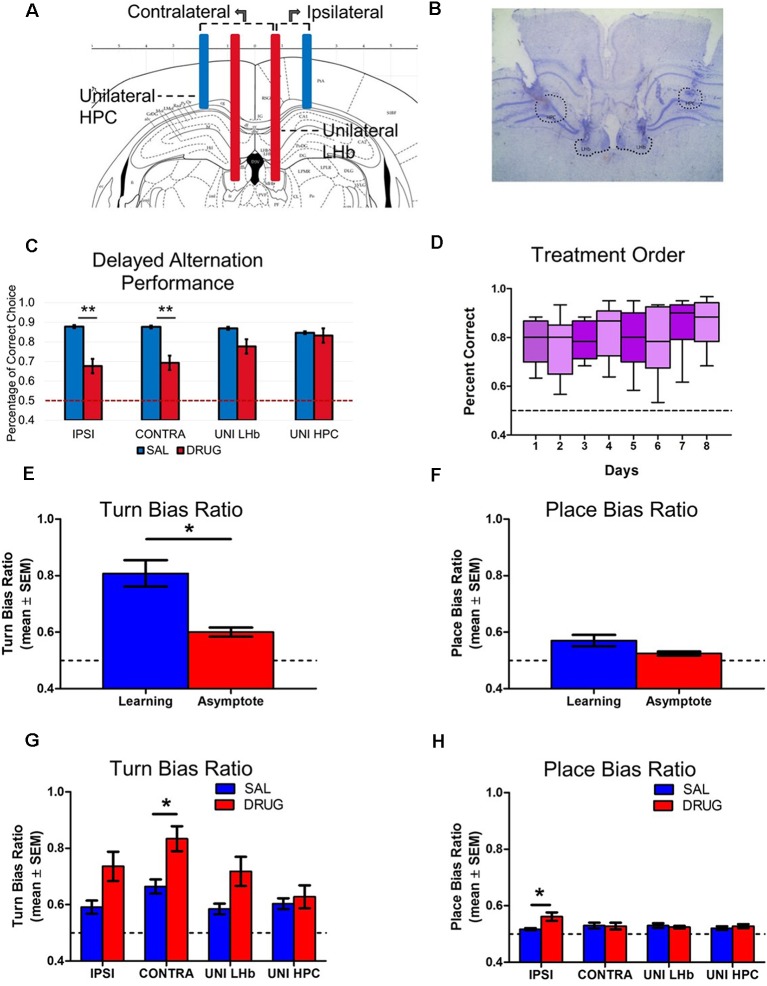
Experiment 2: LHb and HPC disconnection decreases accuracy in a delayed-alternation task.** (A)** Schematic of ipsilateral, contralateral and unilateral infusion cannula placements within HPC and LHb. **(B)** Histological example of cannula placements in the HPC and LHb. **(C)** Any treatment group that impaired both the LHb and HPC showed significant impairments in accuracy (*F*_(3,32)_ = 5.83, *p* < 0.05). **(D)** Results revealed no effect of day of injection on overall accuracy on the delayed alternation task (*F*_(7,64)_ = 0.89, ns.). **(E)** Egocentric turn bias significantly decreased from the Learning phase (0.81 ± 0.04) to Asymptote phase (0.60 ± 0.02; *t*_(8)_ = 5.12, *p* < 0.01). **(F)** The place bias remained at a relatively low level in both Learning and Asymptote phases (Learning = 0.57 ± 0.02, Asymptote = 0.52 ± 0.01) and did not significantly differ from each other (*t*_(8)_ = 1.78, ns.).** (G)** A two-way ANOVA of turn bias scores during asymptotic performance revealed main effects of both drug treatment (*F*_(1,32)_ = 17.91, *p* < 0.01), and injection condition (*F*_(3,32)_ = 13.72, *p* < 0.01), with no interaction effect (*F*_(3,32)_ = 4.39, ns.). Bonferroni *post hoc* tests indicated that there was a significant difference between saline and drug treatment in the contralateral disconnection group (*t*_(8)_ = 3.03, *p* < 0.05). **(H)** A two-way ANOVA of place bias scores did not show main effects for either drug treatment (*F*_(1,32)_ = 2.86, ns.) or treatment area (*F*_(3,32)_ = 1.06, ns.) but did reveal a significant interaction (*F*_(3,32)_ = 3.03, *p* < 0.05). Bonferroni *post hoc* tests indicated that the ipsilateral disconnection group significantly increased place bias scores under drug vs. saline treatment, (*t*_(8)_ = 3.38, *p* < 0.01). *p < 0.05. **p < 0.01. ns = not significant.

#### Experiment 3: Effects of LHb Inactivation on Maze-Based Delay Discounting Performance

##### Habituation and Pre-surgical Training

After maze habituation (as described for Experiment 1), rats were shaped to collect a short delayed small reward (3 s 1 pellet) from either goal arms. Specifically, each rat was placed on the start arm in a given trial and was encouraged to choose one of the goal arms. Upon arrival at the block in front of the reward, the animal had to wait for 3 s before being given access to the reward. Once the rat entered both the LL and SS arm on all following task phases, a block was placed behind the rat to ensure it could not opt out of the choice that was made. The elapsed time was measured by an experimenter using a digital stopwatch. After the block was removed by the experimenter at the termination of the delay, the rat approached and consumed the reward. After the experimenter gently guided the animal to the start arm for the next trial, the food cup was refilled, and the barrier restored. Once the rat was able to finish 16 trials within 20 min, it underwent the surgical implantation of bilateral cannulae.

##### Delay Discounting Task (Figures 5A,B)

After a week of recovery from surgery, all rats were put back on a food-restricted diet. Nine animals underwent delay discounting training in which they learned to choose between a sooner smaller reward (SS; 3 s delay, 1 pellet) and a later larger reward with variable delays (LL; 10, 20, or 40 s delay; 3 pellets). To assess choice preference as a function of delay to LL reward, the three different lengths of delay were tested in separate blocks of trials. That is, each trial block tested only one LL delay interval. The order of the delay blocks were randomly assigned. During the different LL blocks, the SS reward condition remained constant.

Since the rats did not initially know how long they needed to wait for an LL reward, each block began with 10 forced-choice trials followed by six free-choice trials. During the forced-choice trials, five SS and five LL trials were presented in pseudo-random order. For each forced-choice trial, only one goal arm was made available by lowering the other goal arm. Both goal arms were presented during the free-choice trials. These data were used to determine the rats’ choice preference for LL rewards. The three testing blocks were separated by an inter-block interval of 5 min during which the animals were placed on a holding area adjacent to the maze. The location of SS and LL rewards in the goal arms remained constant for each rat but was counterbalanced across rats.

##### Reward Discrimination Task

A reward discrimination task tested rats’ ability to discriminate two goal arms associated with either a small (one pellet) or large (four pellets) reward. Reward discrimination testing took place after infused rats completed the delay discounting test described above. Similar to delay discounting training, rats ran 16 trials (10 forced trials, and six free choice trials). In contrast to delay discounting training, there were equal delays (3 s) between times when rats reached the block and removal of the barrier.

##### Histological Procedures

After completion of all experiments, animals were perfused transcardially with physiological saline followed by 10% formalin. Their brains were extracted and stored in a 10% formalin-30% sucrose solution at 4°C for 72 h. The brains were cut in coronal sections (40 μm) on a freezing microtome. The serial sections were stained with cresyl violet to confirm cannula placements. In Experiment 1, of the initial 27 rats tested, three were removed from the Sal-Sal group, three from the Drug-Sal group, and two from the Sal-Drug group due to cannula outside of the LHb. This meant that final numbers in each group included in the analysis were Sal-Sal = 6, Drug-Sal = 6, and Sal-Drug = 7. Likewise of the 13 animals tested in Experiment 2, 4 were removed due to misplacements. This led to a final number of four males and five females.

## Results

### Experiment 1: LHb Inactivation Impairs Learned Spatial Delayed Alternation but Does Not Alter Initial Learning

Rats with accurate cannula placements (Figure 3F) were tested for the effects of drug or saline injection during the first 3 days of testing on the delayed alternation task. A two-way ANOVA revealed no effect of treatment group (*F*_(2,32)_ = 0.37, ns.), injection day (*F*_(2,32)_ = 2.51, ns.), or an interaction (*F*_(4,32)_ = 1.10, ns.) during this learning phase of the delayed alternation task. One important question was whether drug treatment altered working memory on a trial by trial basis. Because even naive rats perform significantly above chance on the delayed alternation task (demonstrating recall for the previously visited arm), a one-way *t*-test was performed to test whether performance was above chance levels. Results revealed that drug-treated animals (Drug-Sal group) are more accurate than would be expected by chance (*t*_(2)_ = 7.94, *p* < 0.05; Figure 3A).

Following the learning treatments, all groups were then allowed to perform the delayed alternation task without injection until asymptotic performance of three consecutive days of >80% accuracy was achieved. A one-way ANOVA demonstrated no significant differences between treatment groups on days to criterion amongst groups (*F*_(2,16)_ = 0.08, ns.) with days required ranging from (10.83 ± 1.94) in the Drug-Sal group to (12.00 ± 20.5) in the Sal-Drug group.

To examine whether LHb inactivation altered flexible performance of a learned behavior (at asymptotic performance), percent accuracy on the delayed alternation task was examined. A two-way ANOVA revealed a significant effect of treatment group (*F*_(2,32)_ = 30.09, *p* < 0.01), but no effect of injection day (*F*_(2,32)_ = 0.01, ns.), or an interaction (*F*_(4,32)_ = 0.26, ns.) during the asymptotic phase of delayed alternation task testing. Bonferroni posttests demonstrated lower percent correct for the Sal-Drug group across all injection days compared to the Sal-Sal group (*p* < 0.01; Figure 3B). To test for any long-lasting effects of Drug treatment across consecutive days, accuracy on the next day following the final injection was examined. A one-way ANOVA revealed a similar level of performance accuracy following the final day of asymptote treatment (*F*_(2,16)_ = 3.03, ns.) ranging from (80.79 ± 3.32) in the Drug-Sal treatment group to (90.28 ± 1.69) in the Sal-Sal treatment group (Figure 3E).

Rats visited both choice arms roughly equally during learning and asymptotic task phases (Figure 3C). One important question was whether drug treatment altered response strategies during learning and asymptotic performance. This was tested in two ways. First, the likelihood that an animal visited a single reward arm (place bias) during a session was calculated by dividing the number of visits to the most commonly chosen spatial location (per session) by the total number of trials in the session. Thus, a numerical value between 0.5 and 1 represents the proportion of choices for a preferred arm across a session regardless of reward. No effect of treatment group (*F*_(2,16)_ = 0.80, ns.), learning phase (*F*_(1,16)_ = 1.01, ns.), or interaction (*F*_(2,16)_ = 1.55, ns.) was observed with a two-way ANOVA on the ratio of preferred place location over the total number of choices during a session. This result indicates that inactivation of the LHb does not alter the likelihood of a rat to use a place strategy during either task phase.

Rats initially utilized an egocentric turn strategy before executing the correct place alternation (Figure 3D). Similarly, an egocentric turn bias was calculated by dividing the number of egocentric turns (left or right) in the more commonly chosen direction by the total trials in a session. Examination of egocentric turn bias using a two-way ANOVA revealed a significant effect of treatment group (*F*_(2,16)_ = 5.11, *p* < 0.05), learning phase (*F*_(1,16)_ = 38.26, *p* < 0.01), and a significant interaction (*F*_(2,16)_ = 4.23, *p* < 0.05) on the ratio of preferred egocentric turn direction over the total number of choices during a session. Bonferroni posttests showed that the turn bias ratio was significantly elevated in the Sal-Drug group when compared to the Sal-Sal group and the Drug-Sal group selectively during the asymptotic phase of the task (*p*’s < 0.01). This reveals that the Sal-Drug group reinstated a significant turn bias that was common across groups during the learning phase when the LHb was inactivated during asymptote. Rats in the other groups abandon this turn bias in order to successfully obtain reward at above 80% accuracy.

### Experiment 2: LHb and HPC Disconnection Reveals Their Necessary Interaction During Spatial Delayed Alternation

Nine animals with accurate cannula placements (five female and four male rats, Figure 4B) were trained to perform a delayed alternation task on an elevated cross-maze. To investigate the animal’s choice performance under HPC and LHb manipulation, they were tested in eight different injection target (Figure 4A). Before testing, all animals reached continuous asymptotic performance (3 days of >80% accuracy in 60 trials of delayed alternation task).

Our results indicated that any injection target aimed at both the LHb and HPC with drug (ipsilateral and contralateral disconnections) showed significant impairments in accuracy (Figure 4C). Specifically, a two-way ANOVA show significant effects of drug/saline treatment (*F*_(1,32)_ = 46.56, *p* < 0.01) and an interaction (*F*_(3,32)_ = 5.83, *p* < 0.01), but no effect of injection target (*F*_(3,32)_ = 1.96, ns.). Bonferroni multiple comparisons *post hoc* tests indicated a significant difference between saline and drug treatment in the ipsilateral condition (*t*_(32)_ = 5.61, *p* < 0.01), and the contralateral condition, *t*_(32)_ = 5.08, *p* < 0.01. In order to ensure that the order in which rats were given injections across injection days did not alter observed results, a one-way ANOVA of performance accuracy by day of injection was also administered. Results revealed no effect of day of injection on overall accuracy on the delayed alternation task (*F*_(7,64)_ = 0.89, ns.; Figure 4D).

No sex differences in the effects of HPC-LHb disconnection. Because the goal of the present study was the examination of baseline behavior including both sexes and not an examination of sex differences within the behavior, additional animals were not explicitly added to compare sex. Nonetheless, in order to provide information for other researchers and future studies, an underpowered analysis of sex was included to check for trends in behavior. Male and female rats performed in a comparable way in both control and treatment condition. When analyzed by two way ANOVA with sex and drug/saline treatment as factors, the main effect of drug/saline treatment remained (*F*_(3.773,26.41)_ = 12.71, *p* < 0.01) while no effect of sex (*F*_(1,7)_ = 0.00, ns.), or an interaction (*F*_(7,49)_ = 1.117, ns.) was observed.

Place bias is altered with ipsilateral disconnection while contralateral disconnection alters turn bias. As in Experiment 1, preference to select only one reward arm (place bias) or turn preferentially in one egocentric direction (turn bias) was examined during both learning and asymptote. The first 3 days of place bias and turn bias scores during learning were examined using a one-way ANOVA. Results revealed no effect of day on place bias (*F*_(2,16)_ = 2.81, ns.), or turn bias (*F*_(2,16)_ = 0.01, ns.). In order to determine whether a similar pattern of abandoning an egocentric turn bias from initial learning to asymptotic performance (as was observed in Experiments 1 and 2), *t*-tests were performed comparing the first day of learning to the first day of saline treatment during asymptote. Results revealed that, as in Experiment 1, place bias score remained at a relatively low level in both phases (Learning = 0.57 ± 0.02, Asymptote = 0.52 ± 0.01) and did not significantly differ (*t*_(8)_ = 1.78, ns.; Figure 4F). In the control condition, egocentric turn bias did significantly decrease from learning (0.81 ± 0.04) to asymptote (0.60 ± 0.02; *t*_(8)_ = 5.12, *p* < 0.01; Figure 4E). With a similar pattern of choice behavior between both experiments, it raised the question whether disconnection of the LHb and HPC resulted in a similar return to an egocentric turn bias as LHb bilateral inactivation or whether a distinct choice pattern emerged. A two-way ANOVA of turn bias scores during asymptotic performance revealed main effects of both drug treatment (*F*_(1,32)_ = 17.91, *p* < 0.01), and injection condition (*F*_(3,32)_ = 13.72, *p* < 0.01), with no interaction (*F*_(3,32)_ = 4.39, ns.). Bonferroni post tests indicated that there was a significant difference between saline and drug treatment in the contralateral disconnection group (*t*_(8)_ = 3.03, *p* < 0.05; Figure 4G). A two-way ANOVA of place bias scores did not show main effects for either drug treatment (*F*_(1,32)_ = 2.86, ns.) or treatment area (*F*_(3,32)_ = 1.06, ns.) but did reveal a significant interaction (*F*_(3,32)_ = 3.03, *p* < 0.05). Bonferroni post-tests indicated that the ipsilateral disconnection group significantly increased place bias scores under drug vs. saline treatment (*t*_(8)_ = 3.38, *p* < 0.01; Figure 4H).

### Experiment 3: LHb Inactivation Impairs Maze-Based Delay Discounting but Not Reward Discrimination

Nine animals displaying accurate cannula locations (five male and four female rats, Figure 6B) were trained to choose between SS and LL rewards in a delay based decision-making task on an elevated T-maze. To investigate the animal’s choice preference as a function of delay to LL reward, three different delays (10 s, 20 s and 40 s) are used before the delivery of the LL reward in separate blocks of trials. The delay to the SS reward was kept consistent at 3 s for the entire session.

**Figure 5:**
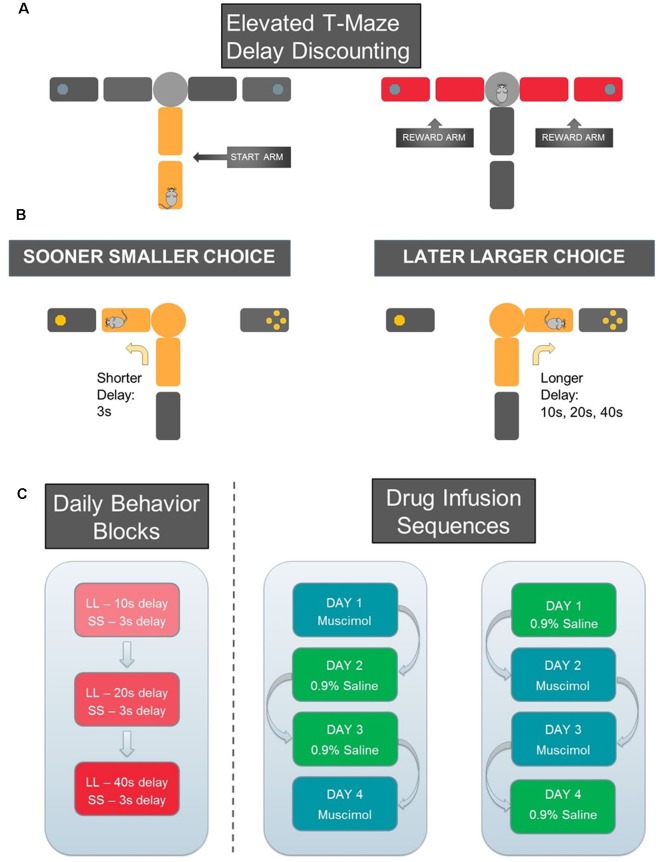
Schematic of Delay-Discounting Task. **(A)** Reward arm (marked in yellow) and two start arms (marked in red) on the elevated T-maze. **(B)** Sooner smaller choice: the animal waited for 3 s to obtain one sugar pellet. Later larger choice: the animal waited for 10 s and obtained four sugar pellets. Once the animal entered one reward arm, the other reward and start arms were blocked by wooden barriers to confine the animal to the chosen arm for the entire delay period. **(C)** For each day of testing, three blocks of trials were offered, each associated with a different length of LL delay (10 s, 20 s, or 40 s). The SS delay remained constant (3 s). The blocks were separated by an inter-block interval of 5 min. Drug infusion sequences follow an ABBA pattern. On day 1 and day 4, a certain animal would receive one type of infusion (0.9% saline or muscimol), and day 2 and day 3 of the alternative type of infusion.

**Figure 6:**
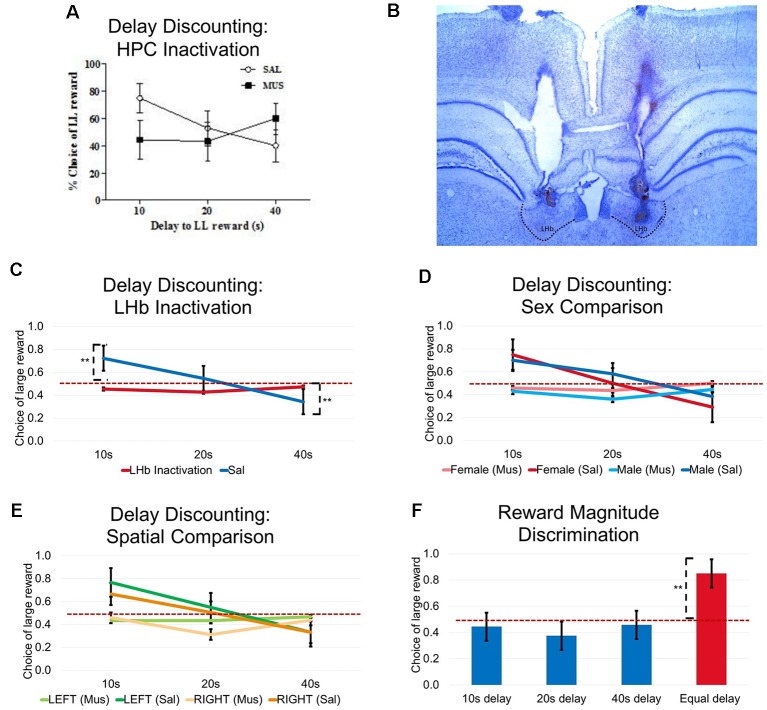
Experiment 3: Bilateral LHb inactivation resulted in impaired performance in delay-discounting task. **(A)** Bilateral HPC inactivation resulted in significantly impaired performance during the delay-discounting task. The figure shown was adapted from Davis et al. (2016). **(B)** Histological illustration of cannula placements in the LHb. **(C)** Bilateral LHb inactivation resulted in a significant impairment in delay-discounting task. There was a significant interaction effect (*F*_(2,16)_ = 34.725, *P* < 0.01), no drug effect (*F*_(1,8)_ = 3.510, *p* = 0.098, ns.), and a significant delay effect (*F*_(2,16)_ = 15.561, *p* < 0.01). **(D)** Impairments were not due to sex differences. No interaction effect was observed after muscimol infusion (*F*_(2,14)_ = 0.206, *p* = 0.816, ns.), and no significant effect of sex was observed (*F*_(1,7)_ = 0.012, *p* = 0.916, ns.). No interaction effects were observed under saline infusion (*F*_(2,14)_ = 0.371, *p* = 0.697, ns.) nor was there a significant sex effect (*F*_(1,7)_ = 0.177, *p* = 0.687, ns.). **(E)** Impairments were not due to a spatial bias toward one or the other reward locatinos. No interaction effect was found after muscimol infusion (*F*_(2,14)_ = 0.127, *p* = 0.882, ns.) and and there was no effect of reward location (*F*_(1,7)_ = 0.057, *p* = 0.819, ns.). No interaction effects were seem after saline infusion (*F*_(2,14)_ = 2.268, *p* = 0.140, ns.) and there was no effect of reward location (*F*_(1,7)_ = 2.356, *p* = 0.169, ns.). **(F)** Intact reward magnitude discrimination was observed after LHb inactivation. Given a consistent delay for either small or large reward choices, animals preferred locations associated with a larger reward, paired sample *t*-test (*t* = 10.513, *p* < 0.01). **p < 0.01. ns = not significant.

Abolished delay discounting behavior following LHb inactivation. During baseline training and SAL control days, rats displayed a strong preference for LL reward over SS reward (choosing LL reward more than 50% of all choices) when the delay to rewards was short (10 s). As delays became longer, they showed a stronger preference for SS rewards. Taking all three delay blocks into consideration, Figure 6C displays a typical delay discounting curve, indicating that rats readily discounted the value of rewards with longer wait times.

By contrast, a repeated measure ANOVA found a significant interaction effect between delay and drug (bilateral Drug injection; *F*_(2,16)_ = 34.725, *P* < 0.01), no drug effect (*F*_(1,8)_ = 3.510, *p* = 0.098, ns), and a significant delay effect, *F*_(2,16)_ = 15.561, *p* < 0.01) such that rats no longer demonstrated a preference for either reward arm regardless of the delay condition. Drug injections resulted in rats choosing both reward arms roughly equally at both the 10 s 20 s, and 40 s delays. SAL injections resulted in normal discounting performance, which animals significantly prefer LL reward at 10 s (Bonferroni posttests, *p* < 0.01) and SS reward at 40 s (Bonferroni posttests, *p* < 0.01) compared to chance levels. This result extends previous findings that the LHb impairs delayed discounting when tested in an operant chamber (Stopper and Floresco, 2014).

As the wooden blocks needed to be manually added and removed, an experimenter was required to be present in the maze room throughout the session. This raises the question of whether the experimenter’s movement could have guided the animal’s choice. This is unlikely as the experimenter always stood in a neutral position near the start arm, equal distance to both reward arm. Furthermore, after the animal finished a trial, the experimenter would bait both arms so as not to bias the rat’s next choice.

No sex differences in delay discounting behavior (Figure 6D). Male and female rats showed similar discounting functions during both baseline and SAL control days. Also, drug injections did not reveal sex differences in the behavioral response to LHb inactivation (no interaction effect under drug infusion: *F*_(2,14)_ = 0.206, *p* = 0.816, ns., and no significant sex effect: *F*_(1,7)_ = 0.012, *p* = 0.916, ns.; no interaction effect under SAL infusion: *F*_(2,14)_ = 0.371, *p* = 0.697, ns., and no significant sex effect, *F*_(1,7)_ = 0.177, *p* = 0.687, ns.). These observations indicate that there were no effects of sex when performing this maze-based flexible response task with or without a normal functioning LHb.

Delay discounting behavior did not depend on a place bias (Figure 6E). In order to exclude the possibility that animals made choices based on the spatial position of the LL or SS arm (as opposed to the subjective evaluation of delay and reward), nine animals (across sex) were randomly assigned to conditions where the right arm always contained the larger reward arm or the left arm contained the larger reward. The choice behavior for the two groups were not different from each other: no interaction effect under drug infusion (*F*_(2,14)_ = 0.127, *p* = 0.882, ns.), and no spatial effect (*F*_(1,7)_ = 0.057, *p* = 0.819, ns.). And there was no interaction effect under SAL infusion (*F*_(2,14)_ = 2.268, *p* = 0.140, ns.), and no spatial effect (*F*_(1,7)_ = 2.356, *p* = 0.169, ns.). This result revealed that the location of the large reward arms did not inadvertently bias the rat’s responses. Rather, their evaluation of the positive value of the reward, and the negative value of waiting, determined their choices.

Intact reward magnitude discrimination after LHb inactivation (Figure 6F). Following the last MUS injection day, after the three delay discounting blocks, rats were trained to discriminate between two goal arms associated with a small (one pellet) and a large (four pellet) reward with only 3 s delays. The results showed that reward magnitude discrimination remained intact after LHb inactivation, as rats preferred the larger reward significantly more often (above 50%) when the delay was constantly short (paired-sample *t*-test, *t* = 10.513, *p* < 0.01). This result shows that LHb inactivation did not ablate the rat’s ability to understand, and make decisions based on, reward value. Rather, it appeared to have disrupted rats’ ability to flexibly respond when task conditions changed.

## Discussion

The current study tested the extent to which the HPC and LHb play similar or discrepant roles in tasks that require flexibly responding to changing task conditions. Using an established hippocampal-dependent delayed alternation maze task, Experiment 1 showed that LHb inactivation impairs spatial delayed alternation performance but only after asymptotic performance was achieved, and not during task acquisition. This finding indicates that LHb is not needed for working memory or spatial identification of goal locations *per se*. Rather, since LHb inactivation impaired choice accuracy after rats reached asymptotic performance levels, it seems to play a specific role in the use of memory to respond flexibly.

The importance of interactions between the LHb and HPC was tested in Experiment 2. The LHb and HPC were disconnected *via* the simultaneous unilateral inactivation of both LHb and HPC experimental paradigm. Disconnecting the LHb and HPC resulted in impaired performance on the delayed alternation task, while choice accuracy was not significantly impaired after only unilateral inactivation of either LHb or HPC. Interestingly the disconnection effect was observed after either ipsilateral or contralateral inactivation of HPC and LHb with the caveat that differences in place and turn bias scores were observed between the ipsilateral and contralateral groups. Previous reports have also observed impairments in ipsilateral and contralateral disconnection of the LHb with the mPFC (Mathis et al., 2016). Since there are no known direct anatomical connections between HPC and LHb, this pattern of results suggests that communication between these structures likely occurs *via* more than one pathway. In either case, the disconnection effect suggests that the LHb and HPC work together to support adaptive and flexible responding, seemingly regardless of the nature of information being processed, and regardless of whether working or reference memory is used.

Experiment 3 tested LHb involvement in another test of response flexibility known to be hippocampal-dependent, maze-based delay discounting (Bett et al., 2015; Davis et al., 2016; Figure 6A). LHb inactivation disrupted delay discounting behavior on our maze: Figure 6C shows that rats no longer adapted choices according to changing delay conditions. Additionally, supplemental tests indicated that the impaired discounting preferences could not be accounted for by changes in motivation, reward magnitude discrimination, absolute reward locations, or sex of the animal. In summary, the combined effects from the three experiments provide further evidence that LHb and HPC likely work together to enable flexible and rapid decision-making in ways that complement the individual spatial and working memory functions of the HPC and mPFC, respectively.

### Complementary Functions Between HPC and LHb

Many studies document the fact that deficits in the habenular region result in impairments in various hippocampal-dependent tasks, and our results are consistent with these findings (Thornton and Davies, 1991; Lecourtier et al., 2004). In Experiment 1, LHb inactivation affected only asymptotic performance in the delayed alternation task, where drug-treated animals displayed an egocentric choice preference (a default strategy only displayed in the early learning phase of the task). This is consistent with the finding that blockade of excitatory inputs to the LHb resulted in impairments of memory retrieval in an HPC-dependent water maze task, which animals exhibited excessive thigmotaxis (default exploratory behavior) under inactivation (Mathis et al., 2015). These results further point out that in contrast to the task acquisition phase, LHb is more critical in the flexible response process after the animal learned the rules required to maximize reward. Matching the findings from Experiment 1, Experiment 2 showed a choice impairment characterized by a return to an egocentric turn bias for contralateral disconnection suggesting that LHb function requires HPC interactions to successfully apply learned rules flexibly. Ipsilateral impairment also impaired choice behavior but while there was a trend for a return to an egocentric choice pattern, it was place bias choices that significantly increased. This raises several interesting possibilities regarding possible differences between information conveyed from a single hemisphere vs. that conveyed *via* both hemispheres. Specifically, it may reflect the somewhat unique lateralized evolutionary path of LHb across species (Ichijo et al., 2017. However, at this point more research is needed on specific input and output pathways between the LHb and HPC to speculate further. The current study clearly supports the view that there is a functional interaction between the two areas when making memory-related flexible response decisions. Such an interpretation is consistent with findings that simultaneous recordings of LHb and dHPC in freely moving rats reveal a subset of LHb cells were phase-locked to hippocampal theta oscillations (Goutagny et al., 2013). In that same study, LHb generated spontaneous theta oscillation that was highly coherent with HPC theta oscillations. The result of Experiment 2 extends our knowledge of the range of behaviors that depend on the coordination between the HPC and LHb.

A previous inactivation study of the role of the LHb in probability-based discounting and delay-based discounting behaviors tested in an operant chamber indicated that LHb impairments suppressed choice biases, making animals indifferent in choosing between rewards correlated to subjective values (Stopper and Floresco, 2014). Expanding this finding, our Experiment 3 adopted a delay discounting task on an elevated T-maze, adding a spatial component to the economic behavioral paradigm. Prior to the current LHb inactivation study, we tested the effects of HPC inactivation on the same maze-based delayed discounting task as that used in here. Identical to the effects of LHb inactivation, HPC inactivation also abolished the delay discounting function when compared to vehicle infusion (Davis et al., 2016). That is, HPC inactivation resulted in animals who were unable to make adaptive choices based on the changing situations, again illustrating that the two structures work together to enable task-specific flexible responding.

### The Behavioral Implementation of HPC and PFC Memory Processing

Previously, studies related to LHb have mostly been focused on depression, fear learning and substance use (Płażnik et al., 1980; Borelli et al., 2005; Sartorius et al., 2010; Maroteaux and Mameli, 2012; Li et al., 2013; Batalla et al., 2017). In the present study, we directly tested the functional interactions between the LHb and HPC during the performance of complex flexible response tasks. Our results are consistent with the model (Mizumori and Baker, 2017) of the LHb as an important integration center between limbic and midbrain monoaminergic systems. For example, anatomical studies confirm that LHb has direct connections to prominent dopaminergic areas (VTA and RMTg) as well as serotoninergic areas (DRN and MRN), both of which importantly contribute to various aspects of behavioral adaptation, including outcome analysis, choice implementation, reward-prediction error perception, reward-risk analysis, reversal learning, monitoring ongoing task process (Conrad and Pfaff, 1976; Aghajanian and Wang, 1977; Pasquier et al., 1977; Graeff and Silveira Filho, 1978; Reisine et al., 1982; Sutherland, 1982; Swanson, 1982; Skagerberg et al., 1984; Wirtshafter and Asin, 1986; Vertes and Martin, 1988; Behzadi et al., 1990; Nagao et al., 1993; Barnéoud et al., 2000; Kim and Lee, 2012; Baker et al., 2015; Balasubramani et al., 2015). The output of the raphe may feed back to HPC as there is a robust efferent projection from the MRN to the HPC (Azmitia and Segal, 1978; Vertes et al., 1999). Such feedback appears to regulate information integration within HPC since disruptions of the MRN generate HPC low-frequency theta activity, while activation of the MRN causes desynchronization of HPC electroencephalographic activity (Maru et al., 1979; Leranth and Vertes, 1999).

According to the Mizumori and Baker model of limbic and midbrain integration *via* the LHb (Baker and Mizumori, 2017; Mizumori and Baker, 2017), the LHb plays a critical and unique role in guiding the behavioral implementation of limbic system memory processing. Briefly, HPC distributes to PFC information regarding the similarity of expected and actual response sequences that result in a desired outcome. This information is integrated into the PFC as it combines HPC input with the multiple other inputs (from e.g., the orbital frontal cortex and striatum) to determine whether the current strategy or responses should continue or whether behaviors should change in order to reach the desired outcome. Following this process, PFC transfers decision signals to efferent targets including the LHb which in turn accommodates internal state information and sensory inputs from LH, SCN and EPN to conclude whether the response decision from PFC is still relevant to the current situation. If internal information indicates that the current response is the optimal to seek the goal, a “match” signal would be transferred to the efferent targets (dopaminergic and serotonergic systems) of LHb to enable continuation of the response. If internal information indicates otherwise, a “mismatch” signal would be sent out to stop or adjust current response resulting in a different behavioral sequence. After the implementation of the choice, LHb could feed back to HPC on the most recent sequence to enhance neural plasticity. As a result, within the HPC, spatially and temporally sequenced memory information can be modified to reflect the developing experience-dependent trajectories.

## Conclusion

The current study provides new and strong evidence that supports the view that the LHb and HPC are part of a functionally connected system that enables flexible responding when choices are driven by changing task conditions. As there is growing evidence that LHb abnormality is related to psychiatric disorders that are characterized by a person’s inability to flexibly switch between learned context-dependent behavioral strategies (Lax et al., 2013; Proulx et al., 2014; Admon and Pizzagalli, 2015), it is important that we better understand how the local circuits within LHb integrates memory and motivational information, and how the result of this integration ultimately directs behavioral responding.

## Data Availability Statement

All datasets generated for this study are included in the manuscript.

## Ethics Statement

The animal study was reviewed and approved by Institute of Animal Care and Use Committee, University of Washington.

## Author Contributions

PB, YR, and SM contributed to the conception and study design. YR, ZR, and EG performed experiments described in this manuscript. PB, ZR, and YR performed data analysis. PB, YR, and SM wrote sections of the manuscript. All authors approved the submitted version.

## Conflict of Interest

The authors declare that the research was conducted in the absence of any commercial or financial relationships that could be construed as a potential conflict of interest.

## References

[B1] Admon R. Pizzagalli D. A. (2015). Dysfunctional reward processing in depression. Curr. Opin. Psychol. 4, 114–118. 10.1016/j.copsyc.2014.12.011 26258159 PMC4525714

[B2] Agetsuma M. Aizawa H. Aoki T. Nakayama R. Takahoko M. Goto M.. (2010). The habenula is crucial for experience-dependent modification of fear responses in zebrafish. Nat. Neurosci. 13, 1354–1356. 10.1038/nn.2654 20935642

[B3] Aghajanian G. Wang R. Y. (1977). Habenular and other midbrain raphe afferents demonstrated by a modified retrograde tracing technique. Brain Res. 122, 229–242. 10.1016/0006-8993(77)90291-8 837230

[B4] Araki M. McGeer P. L. Kimura H. (1988). The efferent projections of the rat lateral habenular nucleus revealed by the PHA-L anterograde tracing method. Brain Res. 441, 319–330. 10.1016/0006-8993(88)91410-2 2451982

[B5] Azmitia E. C. Segal M. (1978). An autoradiographic analysis of the differential ascending projections of the dorsal and median raphe nuclei in the rat. J. Comp. Neurol. 179, 641–667. 10.1002/cne.901790311 565370

[B6] Baker P. M. Mizumori S. J. (2017). Control of behavioral flexibility by the lateral habenula. Pharmacol. Biochem. Behav. 162, 62–68. 10.1016/j.pbb.2017.07.012 28778738 PMC5659956

[B7] Baker P. M. Oh S. E. Kidder K. S. Mizumori S. J. Y. (2015). Ongoing behavioral state information signaled in the lateral habenula guides choice flexibility in freely moving rats. Front. Behav. Neurosci. 9:295. 10.3389/fnbeh.2015.00295 26582981 PMC4631824

[B8] Balasubramani P. P. Chakravarthy V. S. Ravindran B. Moustafa A. A. (2015). A network model of basal ganglia for understanding the roles of dopamine and serotonin in reward-punishment-risk based decision making. Front. Comput. Neurosci. 9:76. 10.3389/fncom.2015.00076 26136679 PMC4469836

[B9] Barker G. R. I. Warburton E. C. (2013). Object-in-place associative recognition memory depends on glutamate receptor neurotransmission within two defined hippocampal-cortical circuits: a critical role for AMPA and NMDA receptors in the hippocampus, perirhinal, and prefrontal cortices. Cereb. Cortex 25, 472–481. 10.1093/cercor/bht245 24035904 PMC4380082

[B10] Barnéoud P. Descombris E. Aubin N. Abrous D. N. (2000). Evaluation of simple and complex sensorimotor behaviours in rats with a partial lesion of the dopaminergic nigrostriatal system. Eur. J. Neurosci. 12, 322–336. 10.1046/j.1460-9568.2000.00896.x 10651887

[B11] Batalla A. Homberg J. R. Lipina T. V. Sescousse G. Luijten M. Ivanova S. A.. (2017). The role of the habenula in the transition from reward to misery in substance use and mood disorders. Neurosci. Biobehav. Rev. 80, 276–285. 10.1016/j.neubiorev.2017.03.019 28576510

[B12] Behzadi G. Kalén P. Parvopassu F. Wiklund L. (1990). Afferents to the median raphe nucleus of the rat: retrograde cholera toxin and wheat germ conjugated horseradish peroxidase tracing, and selective D-[3H]aspartate labelling of possible excitatory amino acid inputs. Neuroscience 37, 77–100. 10.1016/0306-4522(90)90194-9 2243599

[B13] Bett D. Murdoch L. H. Wood E. R. Dudchenko P. A. (2015). Hippocampus, delay discounting, and vicarious trial-and-error. Hippocampus 25, 643–654. 10.1002/hipo.22400 25483408

[B14] Borelli K. G. Gárgaro A. C. dos Santos J. M. Brandão M. L. (2005). Effects of inactivation of serotonergic neurons of the median raphe nucleus on learning and performance of contextual fear conditioning. Neurosci. Lett. 387, 105–110. 10.1016/j.neulet.2005.07.031 16085359

[B15] Brown G. (2018). fill_whole_brain-01Aug17.jpg.Neuroscience and Graphic Design. Available online at: https://neuroscience-graphicdesign.com/portfolio/rat-brain-anatomical-mid-sagittal-plane/. Accessed on June 2, 2019.

[B16] Conrad L. Pfaff D. (1976). Autoradiographic tracing of nucleus accumbens efferents in the rat. Brain Res. 113, 589–596. 10.1016/0006-8993(76)90060-3 953754

[B17] Dalley J. W. Cardinal R. N. Robbins T. W. (2004). Prefrontal executive and cognitive functions in rodents: neural and neurochemical substrates. Neurosci. Biobehav. Rev. 28, 771–784. 10.1016/j.neubiorev.2004.09.006 15555683

[B18] Dalton G. L. Wang N. Y. Phillips A. G. Floresco S. B. (2016). Multifaceted contributions by different regions of the orbitofrontal and medial prefrontal cortex to probabilistic reversal learning. J. Neurosci. 36, 1996–2006. 10.1523/JNEUROSCI.3366-15.2016 26865622 PMC6602019

[B19] Davis J. Sutlief E. Mizumori S. J. (2016). “Hippocampal place fields respond to the expected cost of rewards,” in Poster Presented at SFN 2016, San Diego, CA.

[B20] Goutagny R. Loureiro M. Jackson J. Chaumont J. Williams S. Isope P.. (2013). Interactions between the lateral habenula and the hippocampus: implication for spatial memory processes. Neuropsychopharmacology 38, 2418–2426. 10.1038/npp.2013.142 23736315 PMC3799061

[B21] Graeff F. G. Silveira Filho N. G. (1978). Behavioral inhibition induced by electrical stimulation of the median raphe nucleus of the rat. Physiol. Behav. 21, 477–484. 10.1016/0031-9384(78)90116-6 154108

[B22] Hasson U. Chen J. Honey C. J. (2015). Hierarchical process memory: memory as an integral component of information processing. Trends Cogn. Sci. 19, 304–313. 10.1016/j.tics.2015.04.006 25980649 PMC4457571

[B23] Hikosaka O. Sesack S. R. Lecourtier L. Shepard P. D. (2008). Habenula: crossroad between the basal ganglia and the limbic system. J. Neurosci. 28, 11825–11829. 10.1523/JNEUROSCI.3463-08.2008 19005047 PMC2613689

[B24] Ichijo H. Nakamura T. Kawaguchi M. Takeuchi Y. (2017). An evolutionary hypothesis of binary opposition in functional incompatibility about habenular asymmetry in vertebrates. Front. Neurosci. 10:595. 10.3389/fnins.2016.00595 28101002 PMC5209335

[B25] Kim U. Lee T. (2012). Topography of descending projections from anterior insular and medial prefrontal regions to the lateral habenula of the epithalamus in the rat. Eur. J. Neurosci. 35, 1253–1269. 10.1111/j.1460-9568.2012.08030.x 22512256

[B26] Lax E. Friedman A. Croitoru O. Sudai E. Ben-Moshe H. Redlus L.. (2013). Neurodegeneration of lateral habenula efferent fibers after intermittent cocaine administration: implications for deep brain stimulation. Neuropharmacology 75, 246–254. 10.1016/j.neuropharm.2013.06.034 23891640

[B27] Lecourtier L. Neijt H. C. Kelly P. H. (2004). Habenula lesions cause impaired cognitive performance in rats: implications for schizophrenia. Eur. J. Neurosci. 19, 2551–2560. 10.1111/j.0953-816x.2004.03356.x 15128408

[B28] Leranth C. Vertes R. P. (1999). Median raphe serotonergic innervation of medial septum/diagonal band of Broca (MSDB) parvalbumin-containing neurons: possible involvement of the MSDB in the desynchronization of the hippocampal EEG. J. Comp. Neurol. 410, 586–598. 10.1002/(sici)1096-9861(19990809)410:4<586::aid-cne6>3.3.co;2-8 10398050

[B29] Li K. Zhou T. Liao L. Yang Z. Wong C. Henn F.. (2013). βCaMKII in lateral habenula mediates core symptoms of depression. Science 341, 1016–1020. 10.1126/science.1240729 23990563 PMC3932364

[B30] Mala H. Andersen L. G. Christensen R. F. Felbinger A. Hagstrøm J. Meder D.. (2015). Prefrontal cortex and hippocampus in behavioural flexibility and posttraumatic functional recovery: reversal learning and set-shifting in rats. Brain Res. Bull. 116, 34–44. 10.1016/j.brainresbull.2015.05.006 26033702

[B31] Maroteaux M. Mameli M. (2012). Cocaine evokes projection-specific synaptic plasticity of lateral habenula neurons. J. Neurosci. 32, 12641–12646. 10.1523/JNEUROSCI.2405-12.2012 22956853 PMC6621263

[B32] Maru E. Takahashi L. K. Iwahara S. (1979). Effects of median raphe nucleus lesions on hippocampal EEG in the freely moving rat. Brain Res. 163, 223–234. 10.1016/0006-8993(79)90351-2 218681

[B33] Mathis V. Barbelivien A. Majchrzak M. Mathis C. Cassel J.-C. Lecourtier L. (2016). The lateral habenula as a relay of cortical information to process working memory. Cereb. Cortex 27, 5485–5495. 10.1093/cercor/bhw316 28334072

[B34] Mathis V. Cosquer B. Avallone M. Cassel J. C. Lecourtier L. (2015). Excitatory transmission to the lateral habenula is critical for encoding and retrieval of spatial memory. Neuropsychopharmacology 40, 2843–2851. 10.1038/npp.2015.140 25971591 PMC4864662

[B35] Micheau J. Riedel G. Roloff E. Inglis J. Morris R. G. (2004). Reversible hippocampal inactivation partially dissociates how and where to search in the water maze. Behav. Neurosci. 118, 1022–1032. 10.1037/0735-7044.118.5.1022 15506884

[B36] Mizumori S. J. Baker P. M. (2017). The lateral habenula and adaptive behaviors. Trends Neurosci. 40, 481–493. 10.1016/j.tins.2017.06.001 28688871 PMC11568516

[B37] Mizumori S. J. Yeshenko O. Gill K. M. Davis D. M. (2004). Parallel processing across neural systems: implications for a multiple memory system hypothesis. Neurobiol. Learn. Mem. 82, 278–298. 10.1016/j.nlm.2004.07.007 15464410

[B38] Nagao M. Kamo H. Akiguchi I. Kimura J. (1993). Induction of c-Fos-like protein in the lateral habenular nucleus by persistent noxious peripheral stimulation. Neurosci. Lett. 151, 37–40. 10.1016/0304-3940(93)90039-n 8469434

[B39] Okamoto H. Agetsuma M. Aizawa H. (2012). Genetic dissection of the zebrafish habenula, a possible switching board for selection of behavioral strategy to cope with fear and anxiety. Dev. Neurobiol. 72, 386–394. 10.1002/dneu.20913 21567982

[B40] Pasquier D. A. Kemper T. L. Forbes W. B. Morgane P. J. (1977). Dorsal raphe, substantia nigra and locus coeruleus: interconnections with each other and the neostriatum. Brain Res. Bull. 2, 323–339. 10.1016/0361-9230(77)90066-1 922511

[B41] Płażnik A. Kostowski W. Bidziński A. Hauptmann M. (1980). Effects of lesions of the midbrain raphe nuclei on avoidance learning in rats. Physiol. Behav. 24, 257–262. 10.1016/0031-9384(80)90083-9 6154954

[B42] Proulx C. D. Hikosaka O. Malinow R. (2014). Reward processing by the lateral habenula in normal and depressive behaviors. Nat. Neurosci. 17, 1146–1152. 10.1038/nn.3779 25157511 PMC4305435

[B43] Quina L. A. Tempest L. Ng L. Harris J. A. Ferguson S. Jhou T. C.. (2015). Efferent pathways of the mouse lateral habenula. J. Comp. Neurol. 523, 32–60. 10.1002/cne.23662 25099741 PMC4232452

[B44] Reisine T. Soubrié P. Artaud F. Glowinski J. (1982). Involvement of lateral habenula-dorsal raphe neurons in the differential regulation of striatal and nigral serotonergic transmission cats. J. Neurosci. 2, 1062–1071. 10.1523/JNEUROSCI.02-08-01062.1982 6180148 PMC6564283

[B45] Riedel G. Micheau J. Lam A. G. Roloff E. L. Martin S. J. Bridge H.. (1999). Reversible neural inactivation reveals hippocampal participation in several memory processes. Nat. Neurosci. 2, 898–905. 10.1038/13202 10491611

[B46] Sartorius A. Kiening K. L. Kirsch P. von Gall C. C. Haberkorn U. Unterberg A. W.. (2010). Remission of major depression under deep brain stimulation of the lateral habenula in a therapy-refractory patient. Biol. Psychiatry 67, e9–e11. 10.1016/j.biopsych.2009.08.027 19846068

[B47] Sego C. Goncalves L. Lima L. Furigo I. C. Donato J. Jr. Metzger M. (2014). Lateral habenula and the rostromedial tegmental nucleus innervate neurochemically distinct subdivisions of the dorsal raphe nucleus in the rat. J. Comp. Neurol. 522, 1454–1484. 10.1002/cne.23533 24374795

[B48] Shabel S. J. Proulx C. D. Trias A. Murphy R. T. Malinow R. (2012). Input to the lateral habenula from the basal ganglia is excitatory, aversive, and suppressed by serotonin. Neuron 74, 475–481. 10.1016/j.neuron.2012.02.037 22578499 PMC3471532

[B49] Skagerberg G. Lindvall O. Bjo¨rklund A. (1984). Origin, course and termination of the mesohabenular dopamine pathway in the rat. Brain Res. 307, 99–108. 10.1016/0006-8993(84)90465-7 6087992

[B50] Stephenson-Jones M. Yu K. Ahrens S. Tucciarone J. M. van Huijstee A. N. Mejia L. A.. (2016). A basal ganglia circuit for evaluating action outcomes. Nature 539, 289–293. 10.1038/nature19845 27652894 PMC5161609

[B51] Stopper C. M. Floresco S. B. (2014). What’s better for me? Fundamental role for lateral habenula in promoting subjective decision biases. Nat. Neurosci. 17, 33–35. 10.1038/nn.3587 24270185 PMC4974073

[B52] Sutherland R. J. (1982). The dorsal diencephalic conduction system: a review of the anatomy and functions of the habenular complex. Neurosci. Biobehav. Rev. 6, 1–13. 10.1016/0149-7634(82)90003-3 7041014

[B53] Swanson L. W. (1982). The projections of the ventral tegmental area and adjacent regions: a combined fluorescent retrograde tracer and immunofluorescence study in the rat. Brain Res. Bull. 9, 321–353. 10.1016/0361-9230(82)90145-9 6816390

[B54] Thornton E. W. Davies C. (1991). A water-maze discrimination learning deficit in the rat following lesion of the habenula. Physiol. Behav. 49, 819–822. 10.1016/0031-9384(91)90324-h 1881990

[B56] Vertes R. P. Fortin W. J. Crane A. M. (1999). Projections of the median raphe nucleus in the rat. J. Comp. Neurol. 407, 555–582. 10.1002/(sici)1096-9861(19990517)407:4<555::aid-cne7>3.3.co;2-5 10235645

[B55] Vertes R. P. Martin G. F. (1988). Autoradiographic analysis of ascending projections from the pontine and mesencephalic reticular formation and the median raphe nucleus in the rat. J. Comp. Neurol. 275, 511–541. 10.1002/cne.902750404 3192756

[B57] White N. M. Packard M. G. McDonald R. J. (2013). Dissociation of memory systems: the story unfolds. Behav. Neurosci. 127, 813–834. 10.1037/a0034859 24341707

[B58] Wirtshafter D. Asin K. E. (1986). Discrimination learning and reversal following electrolytic lesions of the median raphe nucleus. Physiol. Behav. 37, 213–219. 10.1016/0031-9384(86)90223-4 3737730

[B59] Yoon T. Okada J. Jung M. W. Kim J. J. (2008). Prefrontal cortex and hippocampus subserve different components of working memory in rats. Learn. Mem. 15, 97–105. 10.1101/lm.850808 18285468 PMC2275661

